# Characterization of the complete mitochondrial genome of *Spheciospongia vesparium* (Demospongiae, Clionaida, Clionaidae)

**DOI:** 10.1080/23802359.2021.2006815

**Published:** 2021-12-10

**Authors:** Hunter J. Rider, Amanda K. MacFarland, Christopher J. Rape, Samantha P. Engster, Viktoria E. Bogantes, Alexis M. Janosik

**Affiliations:** Department of Biology, University of West Florida, Pensacola, FL, USA

**Keywords:** *Spheciospongia vesparium*, sponge, mtDNA, Clionaidae, mitogenome

## Abstract

The Loggerhead sponge (*Spheciospongia vesparium*) is an ecologically important marine species of sponge that provides habitat and food sources to biodiversity hotspots in the Caribbean Sea and along the coasts of Florida. In this study, the complete mitochondrial genome of the sponge, *S. vesparium* was sequenced and reported. The mitochondrial genome of *S. vesparium* was 21,763 base pairs, and consisted of 14 protein-coding genes, 26 tRNA genes, and two rRNA genes. The total nucleotide content comprised 31.01% A, 36.04% T, 11.08% C, and 21.88% G, with a lower GC content of 32.95%. This study provides a phylogenetic analysis of *S. vesparium* and relative sponges in Demospongiae.

Sponges are a diverse clade of filter feeders found in aquatic ecosystems and are ecologically important in cycling nutrients and providing habitat complexity to form biodiversity hotspots (Folkers and Rombouts 2020). The Loggerhead sponge (*Spheciospongia vesparium*, Lamarck, 1815), is a massive species that is distributed throughout the Caribbean Sea and along Florida, U.S.A. coasts. *Spheciospongia vesparium* can be found occupying shallow, soft bottoms of reefs and seagrass beds (Collin et al. 2005). Morphologically, *S. vesparium* is cake-shaped with a flat top that exhibits clusters of holes and a central depression. Although this species has no known commercial value and is toxic to many vertebrates, it houses marine life such as snapping shrimp within its canals (Voss 1976). *Spheciospongia vesparium* is a food source for the endangered Hawksbill sea turtle (*Eretmochelys imbricata*) (Meylan 1988) and coral reef fish species, including Queen angelfish (*Holacanthus ciliaris*), French angelfish (*Pomacanthus paru*), and Rock Beauty angelfish (*Holacanthus tricolor*). Other predators, such as polychaete worms and the red cushion sea star (*Oreaster reticulatus*) consume the Loggerhead sponge (Carballo and Bell 2017). Additionally, through suspension feeding, the Loggerhead sponge has been shown to be helpful in controlling harmful algal blooms of cyanobacteria (Wall et al. 2012). The mitochondrial genome of *S. vesparium* was sequenced to provide a molecular reference for future ecological and evolutionary studies.

*Spheciospongia vesparium* was collected from Apalachee Bay, Wakulla County, Florida, U.S.A. (30°07′25.1″N, 85°43′57.8″W) and preserved in 200 proof ethanol. The specimen was deposited in the Florida Museum of Natural History under voucher number Porifera 004901 (www.floridamuseum.ufl.edu, John D. Slapcinsky, slapcin@flmnh.ufl.edu). Genomic DNA was extracted using the DNeasy blood and tissue extraction kit (Qiagen, Valencia, CA), following the manufacturer's standardized protocol. Library preparation of total genomic DNA was made using the Kapa HyperPlus kit and samples were sequenced using HiSeq Platform, with 250-bp paired-end reads at the Hubbard Center for Genome Studies at the University of New Hampshire (Durham, NH). Raw reads were assembled in Geneious Prime V. 2021.0.3 (https://www.geneious.com) using *de novo* assembly. To infer phylogenetic placement, a maximum-likelihood phylogenetic tree was constructed using MEGA-X (Kumar et al. 2018) with 1000 bootstrap replicates (Figure 1). The mitochondrial genome sequences used in the phylogenetic tree were: MK079942.1 *Cliona varians*; AY320033.1 *Tethya actinia*; NC023834.1; EU237486.1 *Iotrochota birotulata*; NC010171.1 *Negombata magnifica*; MK079949.1 *Phorbas tenacior*; MK079948.1 *Phorbas areolatus*; KR911862.1 *Crella elegans. Polymastia littoralis* (NC023834.1) was used as the outgroup. Annotation of the assembled genome was conducted with MITOS2 (Bernt et al. 2013).

**Figure 1.**
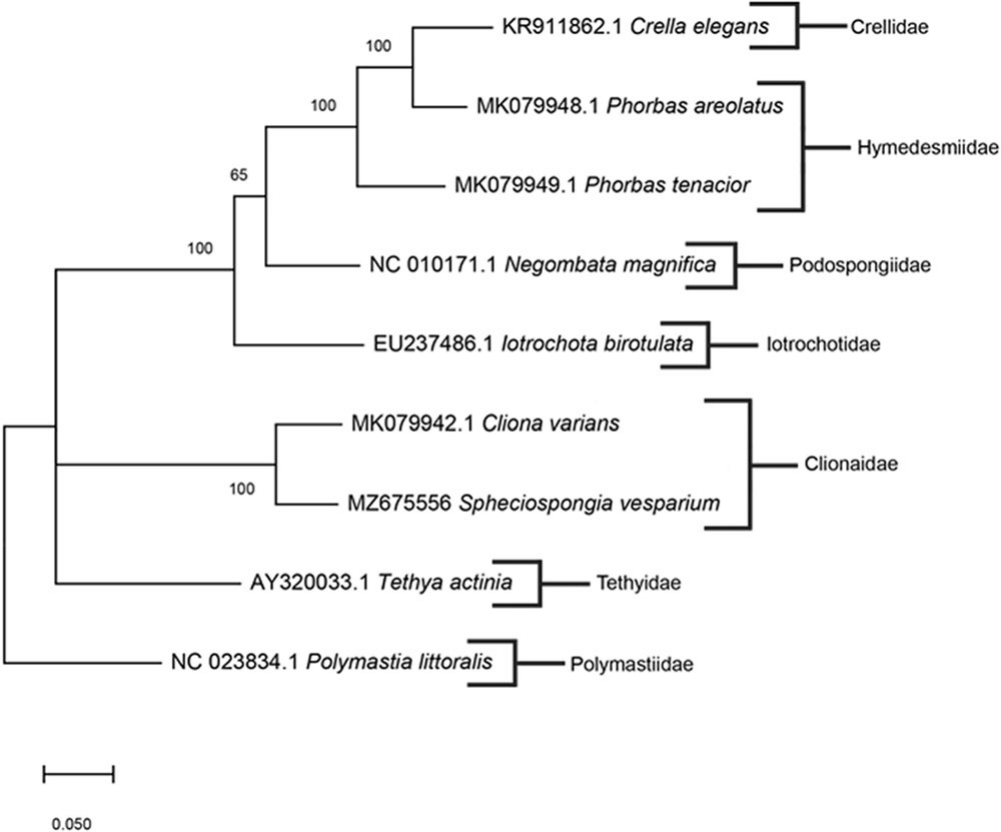
The consensus phylogenetic relationship of *S. vesparium* and other species of Demospongiae based on full mitochondrial genomes. Numbers along the branches specify bootstrap values, and GenBank accession numbers are followed by the species name indicated. Family names are listed to the right of species.

The complete mitochondrial genome of *S. vesparium* was 21,763 bp in length (GenBank accession number: MZ675556) with a base composition of 31.01% A, 36.04% T, 21.88% G, 11.08% C, and had a GC content of 32.95%. This mitogenome contained 42 total genes: two rRNA genes (*rrnL* and *rrnS*); 14 protein-coding genes (*cox1-3*, *atp6*, *8*, *9*; *nad1-6, 4L*, and *cob*); 26 tRNA genes (*trnT, P, H, R, L1, Y, V, C S1, I, S2, L2, M1, L2, E, D, M2, A, F, G, M3, K, S1, Q, W, N*). Additionally, two introns of group I were found in cox1 gene. A high content of non-coding regions and extra genes in the mitochondrial genome of *S. vesparium* is likely due to the retention of several ancestral mitochondrial features throughout metazoan divergence that have been lost in other groups (Lavrov et al. 2005). According to the phylogenetic tree, the closest known relative of *S. vesparium* is *C. varians*. Aside from the outgroup species *P. littorali*s, the phylogenetic tree shows a polytomy between families Tethyidae, Clionaidae, and the rest of the species shown, determining that the tree is partially unresolved and additional data are required. The mitochondrial genome of *S. vesparium* further helps to resolve the phylogenetic limitations of the demosponge group and is beneficial in studying the molecular disparity of sponges.

## Data Availability

The data that support the findings are openly available in NCBI at https://www.ncbi.nlm.nih.gov/, reference number MZ675556. The associated BioProject, SRA, and Bio-Sample numbers are PRJNA731158, SRP320569, and SAMN20122380.
